# Editorial: Bridging the Gap Between Research and Policy in Fostering Social and Emotional Skills

**DOI:** 10.3389/fpsyg.2020.00426

**Published:** 2020-03-25

**Authors:** Javier Suarez-Alvarez, Rubén Fernández-Alonso, Patrick Charles Kyllonen, Filip De Fruyt, Eduardo Fonseca-Pedrero, José Muñiz

**Affiliations:** ^1^Organisation for Economic Co-operation and Development, Paris, France; ^2^Department of Education and Culture, Government of the Principality of Asturias, Oviedo, Spain; ^3^Department of Psychology, University of Oviedo, Oviedo, Spain; ^4^Educational Testing Service, Princeton, NJ, United States; ^5^Department of Developmental, Personality and Social Psychology, Ghent University, Ghent, Belgium; ^6^Department of Education Sciences, University of La Rioja, Logroño, Spain

**Keywords:** social and emotional skills, personality development, social-emotional learning (SEL), cross-cultural, policy research

The world and society are in continuous change, and with them, the skills needed to succeed in life and work (Schleicher, 2018; Burns and Gottschalk, 2019). Aging and more diverse populations, decreasing levels of social and institutional trust, and the dismantling of traditional social networks place additional emphasis on people's sense of trust, cooperation, tolerance, and compassion. The increasing accessibility to information, but also to misinformation such as “fake news,” calls for people's ability to think critically and act independently. Rising complexity and the increasing pace of technological change require the development of self-regulation and metacognitive skills that will enable individuals to monitor and direct their mental processes to become lifelong learners and be able to adjust to rapid changes.

Cognition and emotion are interrelated components of learning, and their interconnected development starts during early infancy and continues throughout the lifespan. Children develop their capacity to experience and express emotions at the same time as they grow physically and cognitively in the development of their language and problem-solving skills (Thompson, 2001). Research evidence shows that social and emotional skills are significant predictors of students' academic performance, such as math and reading, after accounting for socioeconomic status (Chamorro-Premuzic and Furnham, 2008; Suárez-Álvarez et al., 2014). Thus, cognitive development can be impeded when emotions are not well-regulated. Of greater consequence is that although children and adolescents learn at a faster rate than at any other time in their lives, these skills are malleable and change throughout their lifespan (Roberts et al., 2006; Soto, 2015). Indeed, governments are increasingly directing their policies toward enhancing the development of social and emotional skills and they are becoming part of the curriculum and teaching practices in an increasing number of countries.

Over the last years, a large body of research has provided empirical evidence on how to conceptualize, assess, and intervene during the development of social and emotional skills (Abrahams et al., 2019; Kankaraš and Suarez-Alvarez, 2019). However, the assessment of social and emotional skills still faces conceptual and methodological challenges. Moreover, inconsistencies in frameworks, methodology and design, implementation, and administration procedures are potentially leading to highly heterogeneous results when evaluating SEL programs. A recent meta-analysis shows, for example, that higher quality research studies (i.e., randomized experiments) characteristically report smaller effect sizes of SEL programmes than quasi-experiments and smaller studies (Smithers et al., 2018). Ultimately, fostering social and emotional learning relies heavily on combining policy, research, and practice. Therefore, bridging these gaps becomes essential to help policymakers make informed decisions, support teachers in daily practice, and enable children and adolescents to reach their potential.

This article collection, as described below, covers different aspects related to SEL. From policy and practice to psychometric and explanatory modeling, these articles provide alternative paths to address these gaps and point out the challenges and future research in the field. There remains much work to do, but the seven papers included in this article collection can help to mitigate the gaps between research and policy, and advance the field of high quality social and emotional learning research.

Bailey et al. make a reflection of potential unintended consequences of education policy reforms like the “No Child Left Behind Act” in the United States. Components of these policies, such as the increase in student expectations, are intended to have a positive impact on academic achievement. However, these policies often translate into extended time in school, frequent assessments, and policies to strictly manage and control children's behavior. When this occurred, the authors argued that these policies may inhibit children's abilities to build and practice self-regulation skills and jeopardize the relationships between students and teachers. The authors proposed how to shift policies and practices toward developmental science literature.

Aguilar et al. describe the process for creating an international framework for regulating activities and supporting teachers working on social and emotional learning in seven European countries. This study is the result of the successful cooperation between Ministries of Education, NGO's, state institutions and universities across the different education systems. This exploratory study shows specific needs in Spain according to the international framework and discusses the utility of the results for creating new educational policies.

Borgonovi and Pokropek show the importance of attitudes and education in migration trends across 18 countries. After achieving metric invariance for their cross-country comparisons, the results show that better-educated individuals express lower opposition to migration than the poorly educated, and that as much as 60% of education differentials in opposition to migration are due to the mediated effect through feelings of threat.

Kreitchmann et al. provide new evidence on the use of forced-choice questionnaires to mitigate social desirability and acquiesce bias when assessing social and emotional skills, two of the most common response style biases inherent to Likert scales. This research shows that ignoring response style bias in Likert shows the worst validity evidence in comparison to controling the bias through IRT modeling or the use of forced-choice blocks with a multi-unidimensional pairwise preference model (MUPP).

Lozano et al. show how perfectionism (i.e., external pressure, self-exigency, and negative self-evaluation) when well-regulated is associated with emotional development but, when it is not, might be associated with the appearance of clinical symptomatology in childhood. The results show that external pressure and negative self-evaluation are maladaptive dimensions as they predict the appearance of clinical symptomatology. On the other hand, the level of self-exigency acted as a protective dimension favoring the child's positive development.

Álvarez-García et al. analyse risk factors of antisocial behavior in adolescence. The results show that antisocial friendships were a risk factor for antisocial behavior in adolescence, with a moderate effect size. Moreover, while affective and communicative parenting styles were protective factors, parental behavioral control could act as a risk factor for antisocial behavior. Impulsivity and low empathy are mediators of these relationships and show indirect effects between the risk and protector's factors and adolescents' antisocial behavior.

Zhang et al. provide evidence from a meta-analysis of 49 studies about moderators of the relationship between math anxiety and math performance. The math anxiety-performance link was stronger among Asian students than among European/American students. Moreover, this negative link was stronger among senior high school students compared to elementary school students. Finally, this negative link was stronger among studies using custom tests compared to standard tests and studies that assessed problem-solving skills compared to calculation ability.

## Author Contributions

JS-A co-ordinated the Research Topic and drafted the editorial. RF-A, PK, FD, EF-P, and JM provided input during the preparation of the Research Topic, co-edited the manuscripts, and reviewed the editorial.

### Conflict of Interest

The authors declare that the research was conducted in the absence of any commercial or financial relationships that could be construed as a potential conflict of interest.
